# Neural and behavioral traces of error awareness

**DOI:** 10.3758/s13415-020-00838-w

**Published:** 2020-10-06

**Authors:** Hans Kirschner, Jil Humann, Jan Derrfuss, Claudia Danielmeier, Markus Ullsperger

**Affiliations:** 1 Institute of Psychology, Otto-von-Guericke University, D-39106 Magdeburg, Germany; 2 School of Psychology, University of Exeter, Exeter, UK; 3 Donders Institute for Brain, Cognition and Behavior, Radboud University Nijmegen, Nijmegen, The Netherlands; 4 School of Psychology, University of Nottingham, Nottingham, UK; 5 Center for Behavioral Brain Sciences, Magdeburg, Germany

**Keywords:** Error awareness, Post-error adjustments, EEG, Metacognition, Error monitoring

## Abstract

Monitoring for errors and behavioral adjustments after errors are essential for daily life. A question that has not been addressed systematically yet, is whether consciously perceived errors lead to different behavioral adjustments compared to unperceived errors. Our goal was to develop a task that would enable us to study different commonly observed neural correlates of error processing and post-error adjustments in their relation to error awareness and accuracy confidence in a single experiment. We assessed performance in a new number judgement error awareness task in 70 participants. We used multiple, robust, single-trial EEG regressions to investigate the link between neural correlates of error processing (e.g., error-related negativity (ERN) and error positivity (Pe)) and error awareness. We found that only aware errors had a slowing effect on reaction times in consecutive trials, but this slowing was not accompanied by post-error increases in accuracy. On a neural level, error awareness and confidence had a modulating effect on both the ERN and Pe, whereby the Pe was most predictive of participants’ error awareness. Additionally, we found partial support for a mediating role of error awareness on the coupling between the ERN and behavioral adjustments in the following trial. Our results corroborate previous findings that show both an ERN/Pe and a post-error behavioral adaptation modulation by error awareness. This suggests that conscious error perception can support meta-control processes balancing the recruitment of proactive and reactive control. Furthermore, this study strengthens the role of the Pe as a robust neural index of error awareness.

**Electronic supplementary material:**

The online version of this article (10.3758/s13415-020-00838-w) contains supplementary material, which is available to authorized users.

## Introduction

Monitoring for errors is important for successful functioning in daily life. It enables the initiation of remedial actions when something goes wrong and prevents making the same errors over and over again. The ability to monitor and control cognitive processes has been termed metacognition. Metacognition is important to guide our behavior and is well-developed in humans (Fleming, Huijgen and Dolan, 2012; Shea et al., 2014). Sometimes, however, mistakes remain undetected, especially when tasks are complex. In this study, we focus on conscious error perception as a form of metacognition. Research explictily addressing conscious perception of errors, or “error awareness,” has been relatively sparse. The literature concerning the neural signature of error awareness and the role of error awareness in implementing adaptive behavioral adjustments is not unequivocal. We studied the neural and behavioral traces of error awareness in a newly developed number judgement error awareness task. In particular, we focused on the question whether error awareness and its neural correlates modulate the recruitment of various forms of post-error adjustments. First, however, we briefly surveyed the literature on neural correlates of performance monitoring, post error adjustments, and the assessment of error awareness.

### Neural correlates of performance monitoring

The error-related negativity (ERN, or error negativity, Ne) (Falkenstein, Hohnsbein, Hoormann and Blanke, 1990; Gehring, Goss, Coles, Meyer and Donchin, 1993) is a fronto-central negative voltage deflection, peaking 50 to 100 ms after an erroneous response, which seems to be generated in the posterior medial frontal cortex (pMFC) (Debener et al., 2005; Dehaene, Posner and Tucker, 1994; Gruendler, Ullsperger and Huster, 2011; Ullsperger and von Cramon, 2001). Functionally, the ERN is thought to reflect activity of the performance monitoring system after response errors, which is assumed to be conveyed to other brain regions and which implement the necessary adjustments aimed at avoiding errors in the future (Ullsperger, Danielmeier and Jocham, 2014). The underlying mechanisms that give rise to the ERN are still debated. At least two major accounts have been put forward that suggest that the ERN may reflect the detection of post-response conflicts or prediction error signals (for review, see Ullsperger, Fischer, Nigbur and Endrass, 2014).

The association between the ERN and error awareness is not unequivocal. A number of studies found no relationship between ERN amplitude and conscious error perception (Endrass, Franke and Kathmann, 2005; Endrass, Reuter and Kathmann, 2007; Hoonakker, Doignon-Camus and Bonnefond, 2016; Niessen, Fink, Hoffmann, Weiss and Stahl, 2017; Nieuwenhuis, Ridderinkhof, Blom, Band and Kok, 2001; O'Connell et al., 2007; Shalgi, Barkan and Deouell, 2009). Other studies, however, did find the ERN to be dependent on conscious error perception (Maier, Steinhauser and Hübner, 2008; Steinhauser and Yeung, 2010; Wessel, Danielmeier and Ullsperger, 2011), backing up the initial error awareness finding of Scheffers and Coles, who found that the ERN covaried with the perceived inaccuracy of the behavior (Scheffers and Coles, 2000; for a review, see Wessel, 2012).

Similarly, whereas some neuroimaging studies reported that the pMFC was unrelated to error awareness (Hester, Foxe, Molholm, Shpaner and Garavan, 2005; Klein et al., 2007), other studies did find greater pMFC activation when participants were aware of their errors than when they were not aware of an error (Hester et al., 2012; Hester, Nestor and Garavan, 2009; Klein, Ullsperger and Danielmeier, 2013; Orr and Hester, 2012).

The picture is clearer concerning the error positivity (Pe), a positive deflection with centro-parietal scalp distribution following the ERN approximately 200 to 500 ms after the incorrect response (Falkenstein et al., 1990). The Pe is present only when participants were aware of their error (Endrass et al., 2005; Endrass et al., 2007; Nieuwenhuis et al., 2001; O'Connell et al., 2007; Overbeek, Nieuwenhuis and Ridderinkhof, 2005). Murphy, Robertson, Allen, Hester and O'Connell (2012) demonstrated that not only amplitude but also onset of the Pe correlates with the timing of error awareness (i.e., a signaling response of the individual to indicate that the error has been perceived), further promoting the role of the Pe in the emergence of error awareness. Moreover, Boldt and Yeung (2015) showed that, in addition to error detection, the amplitude of the Pe also is associated with the confidence in the error judgment, indicating that these two metacognitive evaluations reflect similar underlying mechanisms (for a theoretical discussion, see Yeung and Summerfield, 2012).

#### Error awareness and post-error adjustments

A question that has not been addressed systematically yet is whether consciously perceived errors lead to different behavioral adjustments compared with unperceived errors. Behaviorally, post-error adaptations are reflected for example in post-error slowing (PES), first described in the 1960s by Rabbitt (1966), post-error reduction of interference (PERI), first described by Ridderinkhof et al. (2002), and post-error improvement in accuracy (PIA) (Danielmeier, Eichele, Forstmann, Tittgemeyer and Ullsperger, 2011; Laming, 1968). Post-error slowing, as the name suggests, describes the phenomenon of prolonged reaction time (RT) in trials following an error compared with trials following a correct response (Rabbitt, 1966). It can be observed in different tasks, but there also are some studies that failed to demonstrate a PES effect (Fiehler, Ullsperger and Von Cramon, 2005). Post-error slowing has been shown to be correlated with pMFC activity as reflected in fMRI studies (Danielmeier et al., 2011; Kerns et al., 2004) and ERN amplitude (Debener et al., 2005; Fischer, Danielmeier, Villringer, Klein and Ullsperger, 2016) during the preceding error trial. Post-error reduction of interference can be observed in interference tasks (e.g., flanker or Simon tasks) and describes a reduction of the interference effect in trials following errors compared to trials following correct responses (King, Korb, von Cramon and Ullsperger, 2010; Ridderinkhof, 2002). The interference effect is the difference in RTs between compatible and incompatible trials. Post-error improvement in accuracy quantifies the improvement of performance after errors, comparing accuracy in trials after errors and after correct responses.

The described post-error adjustments can be classified into reactive and proactive forms of cognitive control (Braver, Gray and Burgess, 2007; Ridderinkhof, Forstmann, Wylie, Burle and van den Wildenberg, 2011). It has been suggested that post-error slowing is a very general response to the rare and salient event of making an error—a reactive adjustment closely associated with the impact of the error that just occurred (Danielmeier and Ullsperger, 2011; King et al., 2010). In contrast, post-error reduction of interference seems to index a proactive control mechanism characterized by preparatory task-set maintenance that enables “early selection” of relevant information, thereby shielding task-relevant information, that guide the correct response, from distracting information or overlearned prepotent response tendencies (King et al., 2010; Ridderinkhof, 2002; Ridderinkhof et al., 2002). While the mechanisms and the adaptivity of different post-error adjustments have been debated in the literature (Fischer, Nigbur, Klein, Danielmeier and Ullsperger, 2018; Purcell and Kiani, 2016; Steinhauser and Andersen, 2019; Ullsperger and Danielmeier, 2016; Wessel, 2018), rather little attention has been paid to how the different modes of control, reactive and proactive, are arbitrated during post-error adjustments. This arbitration of various forms and parameters of cognitive control has been called meta-control (Goschke, 2013; Goschke and Bolte, 2017). It is an unresolved question whether metacognitive functions, such as conscious error perception, have a role in meta-control by balancing the recruitment of proactive and reactive control.

Behavioral post-error adjustments have been less studied under the influence of error awareness than the event-related potentials discussed above. Of the few studies that do exist, some suggest that error awareness is associated with stronger behavioral adjustments, showing that post error slowing is larger after consciously perceived compared to unperceived errors (Cohen, van Gaal, Ridderinkhof and Lamme, 2009; Endrass et al., 2007; Nieuwenhuis et al., 2001; Wessel et al., 2011). Others, however, failed to demonstrate post-error slowing altogether (Klein et al., 2007), or even report post-error speeding (Hester et al., 2005; Hester et al., 2012; Orr & Hester, 2012), but see Danielmeier and Ullsperger (2011) for a more detailed discussion of these seemingly contradicting findings. Klein et al. (2007) reported post-error improvement in accuracy after aware but not after unaware errors, but Endrass et al. (Endrass, Klawohn, Preuss and Kathmann, 2012) found that post-error accuracy is not modulated by awareness. Overall, however, to our knowledge, no studies have addressed all three behavioral post-error adjustments in relation to conscious error perception in a single task.

### Assessment of error awareness

Studies investigating the neural correlates and post-error behavioral adjustments of aware and unaware errors differ in the implemented error signaling procedure and the type of task they used. As reviewed by Wessel (Wessel, 2012), some studies used forced-choice ratings in which participants have to rate their performance as either correct or incorrect (in some studies there also is an additional “don't know” option) after every trial. Other error awareness studies have used an “error signaling button” that only has to be pressed when participants notice an error (Rabbitt, 1968). Such an error signaling button potentially introduces a response bias towards not signaling errors (Ullsperger, Harsay, Wessel and Ridderinkhof, 2010), especially when inter-trial intervals are short. The bin of unaware errors might thus be contaminated by error trials that were not classified as such even though there might have been some residual error awareness. Moreover, only requesting an additional response for aware errors makes it harder to compare them to correct trials that do not require an additional response and unaware errors (i.e., trials which participants do not rate as error and thus do not require an error-button press). Error signaling might thus interfere with trial-by-trial post-error adjustments. Consequently, it is important to keep the procedure as similar as possible for different error types. More recently, it has been proposed that error awareness could be more objectively quantified on the basis of the amplitude of the error positivity (Pe) time-locked to the error-signaling response (Boldt & Yeung, 2015; Murphy et al., 2012).

Finding a task that produces sufficient numbers of both aware and unaware errors is a common problem of error awareness studies. So far, three different kinds of tasks have been used (Klein et al., 2013). First, tasks with degraded (Scheffers & Coles, 2000) or masked stimuli (Cohen et al., 2009; M. Maier et al., 2008; Steinhauser & Yeung, 2010). In such tasks, errors are induced by increased perceptual difficulty and therefore participants are also often uncertain about their performance. Second, antisaccade tasks (Endrass et al., 2007; Klein et al., 2007; Nieuwenhuis et al., 2001; Wessel et al., 2011), in which erroneous prosaccades often are corrected immediately and not noticed. Third, complex tasks, in which a number of competing task rules have to be monitored constantly, and a failure to do so leads to (sometimes unnoticed) errors. The Error Awareness Task (EAT) by Hester and colleagues (Hester et al., 2005) is one example of such a task and has been implemented in a number of studies (Hester et al., 2012; Murphy et al., 2012; O'Connell et al., 2007). The task involves two different Go/NoGo conditions, and participants often have difficulties monitoring both of them at the same time.

So far, complex tasks based on response inhibition like the EAT tended to yield null-findings regarding a relation between the ERN amplitude and error awareness (but see Shalgi and Deouell, 2012), whereas studies that did find effects generally used other tasks (Wessel, 2012). One reason for this could be the error signaling procedure, because studies using the EAT have usually used an error signaling button (Hoonakker et al., 2016; Niessen et al., 2017; O'Connell et al., 2007; Shani Shalgi et al., 2009). Moreover, in contrast to errors in classical choice reaction time tasks like the Eriksen flanker task (Eriksen and Eriksen, 1974), response inhibition errors cannot be corrected and might lead to different neural and behavioral adjustments. It thus seems as if specific types of tasks might lead to specific effects in both electrophysiological and behavioral responses to errors.

### Current study

Taken together, the mixed electrophysiological results and the sparse and somewhat contradictory behavioral results highlight the need for further research to investigate the possible interactions and dependencies between the different error processing components of the performance monitoring network and error awareness. Our goal, therefore, was to develop a task that would enable us to study different neural correlates of error processing (ERN and Pe) and post-error adjustments (PES, PERI, and PIA) in relation to error awareness in a single experiment. Specifically, we were interested in whether the ERN, Pe, and post-error behavioral adjustments are modulated by error awareness and the confidence in this error judgment (quantified by the time participants take to make their judgment). We investigated these associations in a new type of a complex task that does not involve degrading/masking of stimuli or response inhibition and is more similar to classical choice reaction time tasks. Furthermore, we wanted to explore which performance monitoring component is most predictive of error awareness.

## Materials and methods

### Participants

Seventy healthy participants were recruited into this study. The data of seven participants were excluded from analysis, because they either performed the task at chance level (*N* = 1) or did not follow the task instruction, leading to less than 50% valid trials (*N* = 6). The final sample thus consisted of 63 participants (all right-handed, 11 males) in the age range of 18 to 35 years (22.7 ± 3.34 years; mean ± standard deviation [SD]). In a subsample of 32 participants, we recorded EEG data, while participants completed the task (22.7 ± 3.61 years; mean ± SD; 4 male). All participants were informed about the experimental procedures and gave written, informed consent. They were paid by course credits. The study protocol was approved by the local ethics committee.

### Experimental paradigm

**Fig. 1:**
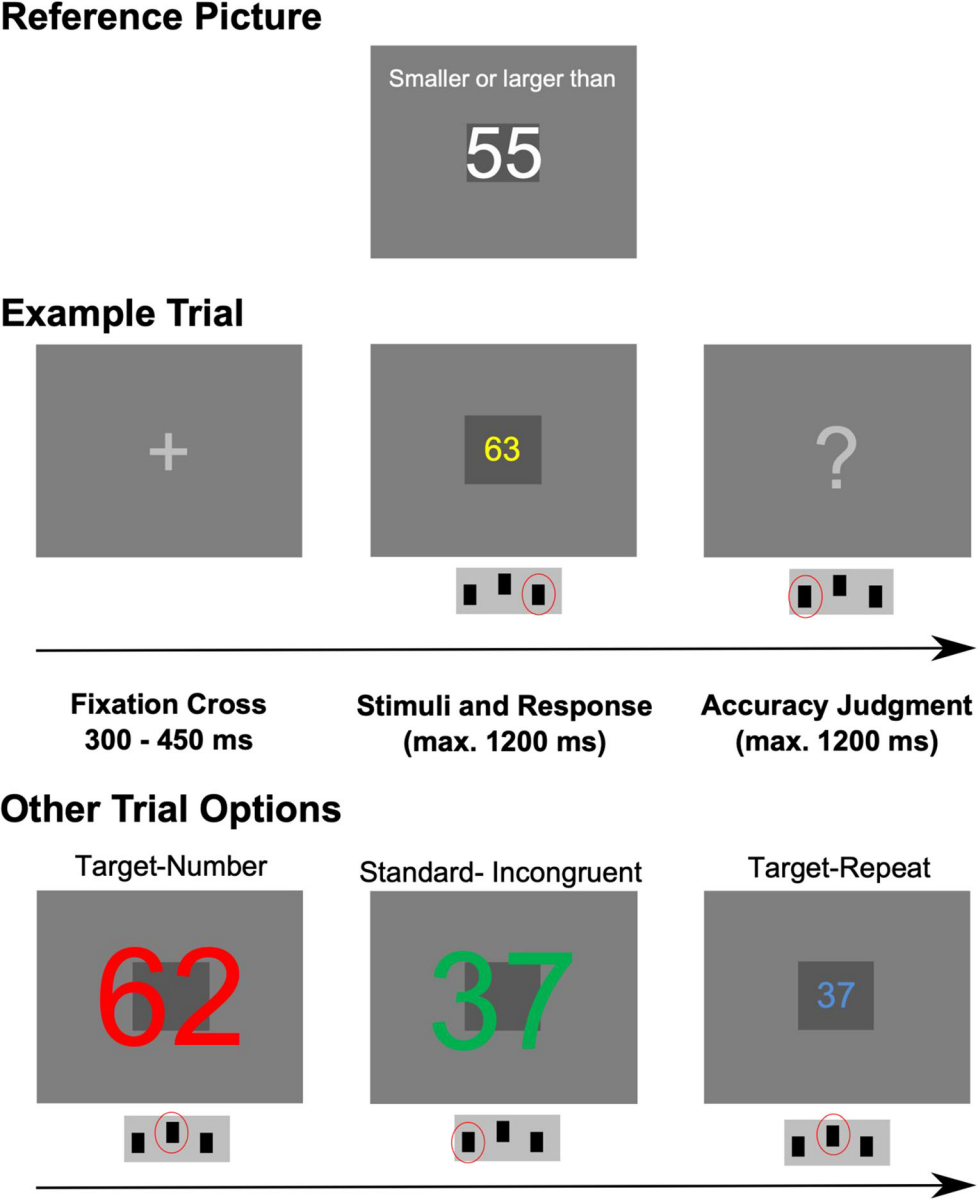
Stimulus layout and trial timing of the number judgment error awareness task. Top row depicts the reference number and frame that was presented once at the beginning of the experiment. Middle row shows an example trial. Bottom row illustrates the other trial options within the task

Participants performed a combined interference and multi-rule target detection task. On each trial, a number between 34 and 76 (except 55) was presented centrally on gray background. Participants were trained to respond by pressing one of three buttons on each trial: Button 1 for all numbers smaller than 55 except 49 (left thumb), Button 2 for all numbers above 55 except 62 (right thumb), or Button 3 for targets (right thumb). Targets were trials where the presented number was equal to the preceding number (repeat target), or where the numbers 49 or 62 appeared (number targets) (Figure 1).

The numbers were presented in different colors (red, yellow, green, or blue) and small or large font sizes (vertical visual angles of 1.9 and 9.5° at a screen distance of 60 cm), but these features were task-irrelevant. We used different colors to make it harder to detect repeat-targets, and different font sizes to manipulate congruency. Trials were congruent when the physical size (big or small font size) matched the magnitude (higher or lower than 55) of the number, and incongruent if not. Moreover, to make (in)congruency more salient, we introduced a reference frame (dark grey square behind the number) at the beginning of the experiment: The reference number 55 (white on dark reference frame) was presented once in an intermediate font size so that the number had exactly the size of the reference frame. During the experiment, the reference frame was presented in the background behind the stimuli, so that the physical number size was clearly smaller or larger than the reference frame (Figure 1).

After each response, participants had to indicate whether they thought they responded correctly or not by pressing either the left button (subjective correct response) or right button (subjective error). We rated errors followed by a right button press as aware errors and errors followed by a left button press as unaware errors.

Depending on a random temporal jitter, stimulus presentation started at 300, 350, 400 or 450 ms into every trial. Stimuli were presented up to 1,200 ms. During this time-window, the motor response had to be made. After participants’ responses, the screen cleared for 500 ms. Then, participants were presented a question mark, indicating that they had to make their accuracy judgment. There was a 1,200-ms deadline for this decision. Immediately after participants’ accuracy judgments, the next trial started. Hence, total trial duration was maximal 3,200 to 3,350 ms. The experiment comprised 1,000 trials (50% incongruent), consisting of 800 standard trials requiring a smaller/bigger than 55 judgment, 100 number target trials, and 100 repeat target trials. Trial order was pseudo-randomized, ensuring that two target trials were never presented subsequently and that transitions between congruent and incongruent trials were counterbalanced. Trials were presented in 10 blocks of 100 trials, with a short self-paced break in-between blocks. Before starting the experiment, participants performed a practice session comprising 20 trials. Speed and accuracy of the response were equally emphasized in the task instruction. Stimuli were presented with Presentation 20.2 (Neurobehavioral Systems, San Francisco, CA) on a 22-inch monitor with a resolution of 1920 x 1,200 pixels.

This new number judgment error awareness task has several advantages to tasks commonly used in the literature (e.g., the EAT (Hester et al., 2005)). First, this task is more similar to classical choice reaction time tasks by introducing interference effects, which potentially allow us to study post error adaptations, such as post-error reductions of interference. Second, using a forced-choice error signaling procedure at the end of every trial ensured that trials after aware errors did not differ from trials after unaware errors or after correct responses, therefore making different types of post-error trials more comparable to each other and to post-correct trials, enabling post-error slowing to be calculated in comparable trials. In addition, not having an error signaling button, as also is used in the original EAT, prevents us from potentially introducing a response bias toward not signaling errors (Ullsperger et al., 2010). Third, this task does not involve degrading/masking of stimuli or response inhibition thereby enabling the participant to adapt behavior after the commission of errors.

### EEG acquisition and processing

An elasticated Easycap EEG cap with a montage that placed 61 Ag/AgCl sintered electrodes on five concentric rings equidistantly spaced around Cz and BrainAmp MR plus amplifiers (Brain Products) were used to record EEG. The positions of the electrodes on each ring also were equidistant from each other, and the vertical and horizontal central lines were identical to the positions of the 10% system. Data were recorded at a sampling rate of 500 Hz. Impedances were restricted to below 5 kΩ. Vertical and horizontal eye movements were recorded with channels placed above and below the left eye and on the outer right and left canthi, respectively. The ground electrode was located between Fz and AFz and the reference electrode at CPz. We analyzed all data under Matlab 2017b (The MathWorks, Natick, MA) and the EEGlab 13 toolbox (Delorme and Makeig, 2004) using custom routines.

After data acquisition, the EEG data were filtered with a 0.5 Hz high- and 42 Hz low-pass filter and re-referenced offline to the common average (i.e., finding the arithmetic mean across all electrodes sites and then subtracting this value from each site). The continuous data were then segmented into stimulus-locked epochs from 500 ms pre-stimulus to 2,500 ms post-stimulus. Epochs that contained artifacts were automatically rejected based on joint probabilities (e.g., epochs containing deviations greater than a specified threshold of the mean probability distribution of trials were rejected, the starting threshold was 4.5 SD), yet no more of 10% of the trials were excluded as otherwise the rejection threshold was increased (in this sample the threshold remained the starting threshold of 4.5 SD). On average 48.03 (SE = 3.19; min = 15; max = 81) epochs have been removed from the data set. Data were then demeaned and decomposed into independent components with the extended runica infomax ICA algorithm of Bell and Sejnowski (1995) implemented in EEGLab. The time courses and topographies of the independent components of each dataset were visually inspected for components reflecting eye blinks, horizontal eye movements or electrode artifacts, and those components were removed from the data (mean [M] = 5.72; standard error [SE] = 0.34; Min = 2, Max = 12). Thereafter, data were re-segmented into response-locked epochs from 500 ms pre-response to 1,000 ms post-response. Following baseline correction (−200 to −50 ms relative to response onset), the data were then used for multiple robust single-trial regression analyses (Fischer et al., 2016; Fischer and Ullsperger, 2013).

### Data analysis

#### Behavioral analyses

Trials with reaction times below 80 ms were removed from the datasets (standard trials: M = 0.59 (SE = 0.19; Min = 0, Max = 11); target trials: M = 0.10 (SE = 0.04; Min = 0, Max: 1)). Moreover, trials with or following invalid responses (e.g., multiple responses and misses; standard trials: M = 55.25 (SE = 5.95; Min = 4, Max = 244); target trials: M = 12.24 (SE = 1.37; Min= 0, Max = 47)) or accuracy judgments were also excluded (standard trials: M = 20.22 (SE: 3.41; Min = 1, Max = 165); target trials: M = 10.62 (SE = 3.0287; Min = 0, Max = 162)). Next, we determined critical factors that influence RT and accuracy in the task in two multiple robust regression models using each participant’s single-trial log-scaled RT and accuracy to evaluate participants task performance.

The RT model consisted of the following regressors:

$$ \log (RT)={b}_0+ Trial\ Type\times {b}_1+ Congruency\times {b}_2+ Jitter\times {b}_3+ Distance\times {b}_4+ Confidence\times {b}_5+ Trial\  Nr\times {b}_6+e $$

RT GLM

The logistic accuracy model was defined by:

$$ Accuracy={b}_0+ Trial\ Type\times {b}_1+ Congruency\times {b}_2+ Jitter\times {b}_3+ Distance\times {b}_4+ Confidence\times {b}_5+\mathit{\log}(RT)\times {b}_6+ Trial\  Nr\times {b}_7+e $$

Accuracy GLM

The individual factors of these models were defined as follows: *Trial Type* (−1 standard, 1 = target), *Congruency* (−1 = congruent, 1 = incongruent), *Jitter* (−1 = short (300 and 350 ms), 1 = long (400 and 450 ms)), *Distance* = the absolute numerical distance between the presented number and the reference number, *Confidence* = the log-scaled time participants took to make their subjective error judgment in the previous trial, *TrialNr* = the log-scaled current trial number (reflecting the time in the task). TrialNr served mainly to control for unspecific effects of task duration, like changes in motivation, fatigue, or response caution.

To investigate post-error adjustments and their modulation by error awareness within the task, while controlling for possible confounds, we included the regressor previous accuracy (previous correct = −1, previous incorrect = 1) to the RT and accuracy models. The models investigating post-error adaptations after aware errors comprised trials that either followed correct responses or consciously perceived errors. Whereas the models investigating post-error adaptations after unaware errors were run on trials following correct responses and post unaware errors trials. Post-error slowing and post-error increases in accuracy were investigated by comparing trails that followed correct responses to trials that either followed aware or unaware errors within the previous accuracy regressor in the respective model. Post-error reductions in interference were investigated within the interaction-term between the previous accuracy and congruency regressor of the respective model. To explore, whether post-error slowing was depending on the temporal jitter (e.g. the time to prepare for the primary task after the accuracy judgment), we included the interaction term previous accuracy x jitter to the respective model. Moreover, given that interference in the task could also be induced by the numerical distance between the presented number and the reference number on a given trial, we investigated whether this effect is modulated by the previous accuracy within the previous accuracy x distance regressor in the respective model. Note that the RT GLMs were calculated on both current correct and current error trials.

Within-participant regression weights were tested for significance using two-sided t-tests. Individual participants’ t-values per regressor were then tested on group level via two-sided t-tests against zero corrected for multiple comparisons (0.05/number of regressors). We followed up regression effects by binning the data according to significant factors.

#### Single-trial EEG analyses

For the EEG analyses, we applied a multiple robust single-trial regressions approach to identify trial-by-trial fluctuations in the EEG signal in response to specific factors in our task (Fischer et al., 2016; Fischer & Ullsperger, 2013). These analyses were conducted using custom code written in Matlab 2017b (The MathWorks, Natick, MA) and the EEGlab 13 toolbox (Delorme & Makeig, 2004). We focused on two components that are typically seen after erroneous responses, the ERN and Pe (Ullsperger, Fischer, et al., 2014).

In the first generalized linear model (EEG GLM 1), we investigated whether general error processing is reflected in the neural data. Apart from the regressor Accuracy, this model included the following regressors to account for possible confounds: Current log-scaled RT; Congruency (congruent/incongruent); Distance of the current number to the reference number; log-scaled Trial Number.

$$ \mathrm{EEG}={b}_0+ Error\times {b}_1+ Distance\times {b}_2+\mathit{\log}(RT)\times {b}_3+ Congruency\times {b}_4+ Distance\times {b}_5+ Trial\  Nr\times {b}_6+e $$

EEG GLM 1

In a second model (EEG GLM 2), we investigated whether the ERN and Pe on error trials was modulated by error awareness and the confidence in the subjective error judgment. Therefore, we included the following factors: Error Awareness (aware/unaware error), Confidence, and the interaction between Error Awareness and Confidence. Moreover, this model included as regressors to account for possible confounds: Current log scaled RT; Congruency (congruent/incongruent); Distance of the current number to the reference number; Trial Number. We chose the electrode site with the maximal effect of EEG GLM 1 as target for this analysis.

$$ \mathrm{EEG}={b}_0+ Error\ Awareness\times {b}_1+ Confidence\times {b}_2+ Error\ Awareness\ Confidence\times {b}_3+ Distance\times {b}_4+\mathit{\log}(RT)\times {b}_5+ Congruency\times {b}_6+ Trial\  Nr\times {b}_7+e $$

EEG GLM 2

These analyses resulted in regression coefficients for every time point and electrode, revealing the time course and scalp topographies of the relationship between each predictor and neural activity. We corrected for the number of regressors in these analyses and report *p* values with this correction applied. Please note, that trials excluded from the behavioral analyses due to too fast responses and invalid responses also were removed from the EEG analyses.

#### Multivariate pattern analyses

We used single-trial neural activity (epochs spanning from −100 ms to 500 ms after erroneous responses) of the whole scalp to train a support vector machine to classify if a participant consciously perceived an error on a given trial. Therefore, we applied the support vector machine functions implemented in MATLAB 2017b (fitcsvm, predict). The neural data was averaged −10 ms to +10 ms around each datapoint with a step size of 10 ms throughout each epoch. All input data was z-scored across and within electrodes and time. A 50-fold cross-validation using 90% of the trials as training and 10% of the trials (but at least 10 trials) as prediction set was applied. Accuracy was calculated as the percentage of overlap between predicted labels and the ground truth (i.e., participants error awareness) at each datapoint and for each participant. Available trials for aware and unaware errors were matched with a reduction to the smaller data size via random subsampling. To localize the information for the classification, we applied a searchlight analysis approach (Fischer et al., 2016) using the same settings as described above. This resulted in an average accuracy per electrode and time point. We report the average peak accuracy and topography of these results. To establish if the peak accuracy was statistically above chance level, we applied a permutation test with 50,000 iterations.

#### Intertrial phase clustering

To explore, whether latency variability could potentially contribute to the differences seen in EEG analyses, we calculated the intertrial phase clustering (ITPC) as a measure of the consistency of time-frequency phase angles over trials (Cohen, 2014). We applied the following formula to calculate ITPC and followed the procedures described in Cohen (2014). The description is adapted from therein.

$$ ITPC=\left|{n}^{-1}\sum \limits_{r=1}^n{e}^{ik_{tfr}}\right| $$

Here, **n** is the number of trials; **n**^**-1**^ combined with the summation operator indicates an average. **E**^**ik**^ is from Euler’s formula and provides the complex polar representation of a phase angle **k** on trial **r,** at time-frequency point **tf**.

To control for differences in trial count between aware and unaware errors we Rayleigh’s Z transformed the intertrial phase clustering with the following formula:

$$ {ITPC}_z=n\ast {ITPC}^2 $$

Here, **n** is the number of trials. Higher values indicate higher phase clustering.

## Results

### Behavioral results

On average, participants performed the task with an error rate of 12.74% (standard error [SE] = 0.74; *N* = 115.60 ± 6.72 trials). Of these error trials, 57.83% (SE = 2.14) were consciously perceived as errors, whereas 42.16% (SE = 4.05) of the errors remained unnoticed. This difference was significant (t(62) = 2.84, *p* = 0.006, 99% confidence interval [CI] [4.48, 25.71]). Moreover, we found a difference in RT between reported errors versus unreported errors (332.39 (SE = 13.10) ms vs. 293.32 (SE = 14.02) ms, t(62) = 3.79, *p* < 0.001, 99% CI [18.17, 59.65]). In addition, participants classified 92.69% (SE = 2.81) of their correct responses and 7.31% (SE = 2.80) of their erroneous responses as correct. Taken together, these results indicate that the task is suited to study error awareness, because it yielded both a sufficient amount of aware and unaware errors, and that the accuracy judgments were above chance level.

**Fig. 2:**
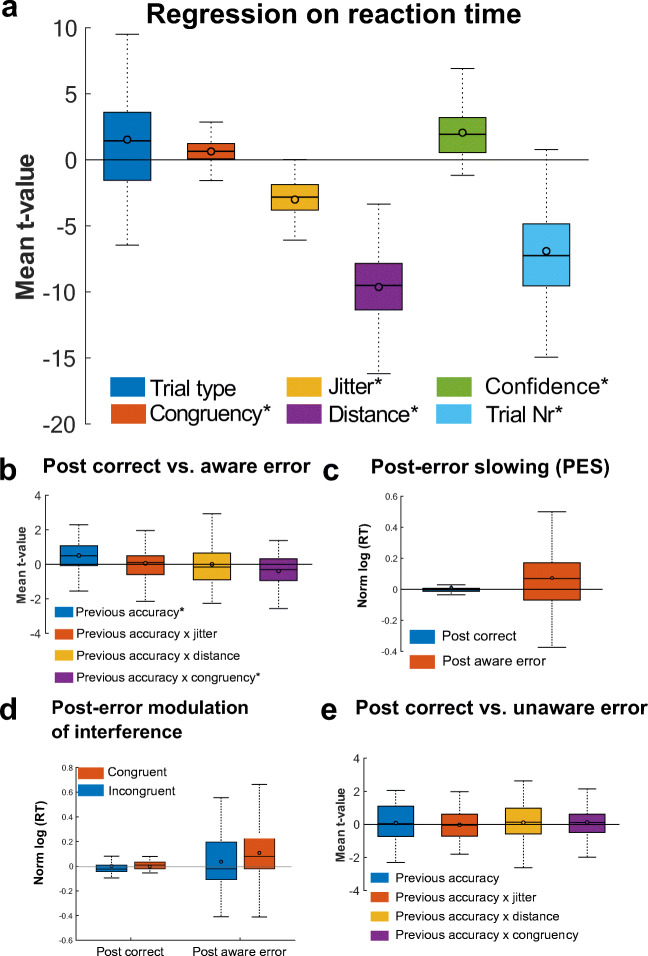
Results of the RT regression model. **A** Multiple single-trial regression on RT was used to evaluate general task behaviour. The results in our number judgment error awareness task are typical for classical choice reaction time tasks and reflected effects of interference and the interval between the last response and next on participants. Results suggested PES was only observed after aware errors (**B**, **C**). Additionally, we found a modulation of the interference effect after aware errors (**D**). **A**, **B**, and **E** display averaged within subject *t*-values. *Significant regressor (derived from *t*-tests of the individual regression t-values against zero). Boxes = interquartile range (IQR), − = median, o = mean, whiskers =1.5 × IQR. *N* = 5 participants were excluded, because no model fit was found

**Fig. 3:**
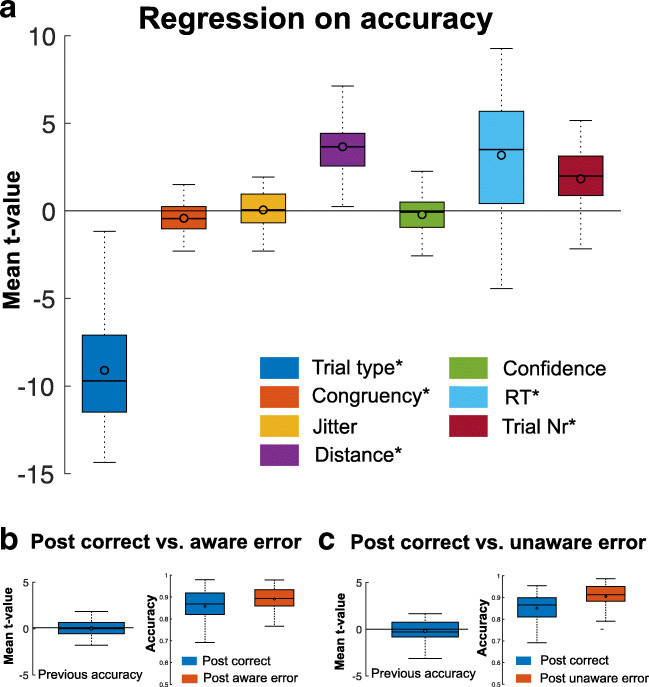
Results of the logistic Accuracy regression model. **A**) logistic regression on accuracy. Although, descriptively there was an increase in accuracy after both aware (**B**) and unaware errors (**C**), when controlling for other factors influencing accuracy on a given trial these effects did not reach significance. *Significant regressor (derived from *t*-tests of the individual regression *t*-values against zero). Boxes = interquartile range (IQR), − = median, o = mean, whiskers =1.5 × IQR. *N* = 6 participants were excluded because no model fit was found

To disentangle specific task-related effects and to investigate post-error adjustments in their relation to error awareness, we determined critical factors influencing RT and accuracy in multiple robust regression models, using each participant’s single-trial RT and accuracy. Results are presented in the following sections and in Figures 2 and 3.

#### Evaluation of participants behaviour in the task

An overview of the results of the RT and accuracy GLM are depicted in Figures 2A and 3A. First, regression analyses revealed that on target trials, there was a trend for participants to respond slower (ΔRT = 28 ms, *t*(57) = 2.56, *p*(corrected) = 0.078, 99% CI [0.33, 2.73]) and a strong effect to commit more errors (ΔAccuracy = 32%, *t*(56) = -20.73, *p*(corrected) = 4.24 x 10^-27^, 99% CI [−9.95, −8.19). Second, we confirm that incongruence increases RT (ΔRT = 5 ms, *t*(57) = 4.97, *p*(corrected) = 3.867 x 10^-5^, 99% CI [0.38, 0.89]) and decreases accuracy (ΔAccuracy = 1%, *t*(56) = −3.36, *p*(corrected) = 0.01, 99% CI [−0.67, −0.17]), demonstrating the expected congruency effect. In addition, the duration of the jitter influenced RT, with longer RT for a shorter Jitter (ΔRT = 27 ms, *t*(57) = −14.01, *p*(corrected) = 2.49 x 10^-19^, 99% CI [−3.44, −2.58]). Moreover, RT and accuracy were influenced by the numerical distance of the presented number to the reference number, with longer RT (*t*(57) = −24.74, *p*(corrected) = 2.1499 x 10^-31^, 99% CI [−10.40, −8.85]) and lower accuracy (*t*(56)= 14.18, *p*(corrected) = 2.42 x 10^-19^, 99% CI [3.14, 4.17]) on trials where the presented number was closer to the reference number. Further, confidence in the previous accuracy judgment, operationalized as the time a participant took to decide whether or not they made a mistake in the last trial, modulated RT in the consecutive trial, whereby less confidence (i.e., longer accuracy judgment times) was associated with prolonged RTs in the following trial (*t*(57) = 7.8951, *p*(corrected) = 6.16 x 10^-10^, 99% CI [1.54, 2.58]). Finally, shorter RTs were associated with lower accuracy, reflecting a speed-accuracy trade-off (*t*(56) = 8.14, *p*(corrected) = 3.17 x 10^-10^, 99% CI [2.34, 3.87]).

Taken together, the results in our number judgment error awareness task are typical for classical choice reaction time tasks and reflect effects of interference and the interval between the last response and the next stimulus on participants’ behavior. Moreover, trial type (standard vs. target) had an influence on accuracy and RT. This suggests that the task is suitable to investigate post-error adaptions. A detailed visualisation of the results of the RT and accuracy model can be found in the supplementary materials.

#### Behavioral post-error adjustments

##### Post-error slowing (PES)

To investigate whether error awareness had an effect on post-error slowing over and above other factors influencing RT on a given trial, we included previous accuracy into the RT model and compared trials following correct responses with trials either following aware and unaware errors. The results of these models are depicted in Figure 2 and revealed that, while controlling for possible confounds, PES is only present after aware errors (ΔRT = 11.5 ms, *t*(57) = 3.63, *p*(corrected) = 0.004, 99% CI [0.23, 0.79], see Figures 2B & C), but not after unaware errors (*t*(57) = 0.55, *p*(corrected) = 1, 99% CI [−0.20, 0.36]; Figure 2E). The post-error slowing effect was not depending on the jitter (e.g., the time to prepare for the primary task after the accuracy judgment; Figures 2B and E).

##### Post-error reduction of interference (PERI)

A significant interaction between previous accuracy and incongruence confirmed that the incongruence effect is modulated by previous accuracy and that this effect is only present on trials following aware errors (*t*(57) = −2.97, *p*(corrected) = 0.029, 99% CI [−0.64, −0.12]; Figures 2B and D), but not after unaware errors (*t*(57) = 0.85, *p*(corrected) = 1, 99% CI [−0.16, 0.41]; Figure 2E). However, our data suggest an increase in interference, which was reflected by a larger difference between congruent and incongruent trials after aware errors (ΔRT = 12.5 ms) compared with trials following correct responses (ΔRT = −4.2 ms). There was no modulation of the distance regressor by previous accuracy (Figures 2B and E).

##### Post-error increases in accuracy (PIA)

Participants displayed a descriptive increase in accuracy on trials following both aware (3.31%; Figure 3B) and unaware errors (5.34%; Figure 3C). However, when controlling for possible confounds, this effect did not reach significance (corrected *p* = 1 for both error types; Figures 3B and C).

Because the same effectors were used for task responses and error awareness judgment, we investigated whether there was a systematic relationship between the task response, the response related to error awareness (right: error, left: correct), and error awareness RT, that could potentially have biased our behavioral results. We did not see any systematic relationship (all r < 0.2, see *Supplementary Materials*). Moreover, the behavioral results could have been biased, because the accuracy judgment responses (right: error, left: correct) were not counterbalanced across participants. However, there was no RT difference between left and right responses in the primary task responses (t(62) = 1.38, *p* = 0.17, 99% CI [−2.43, 15.60]), making a systematic response bias in accuracy judgment less likely.

### Electrophysiological results

#### Error-related EEG activity

**Fig. 4:**
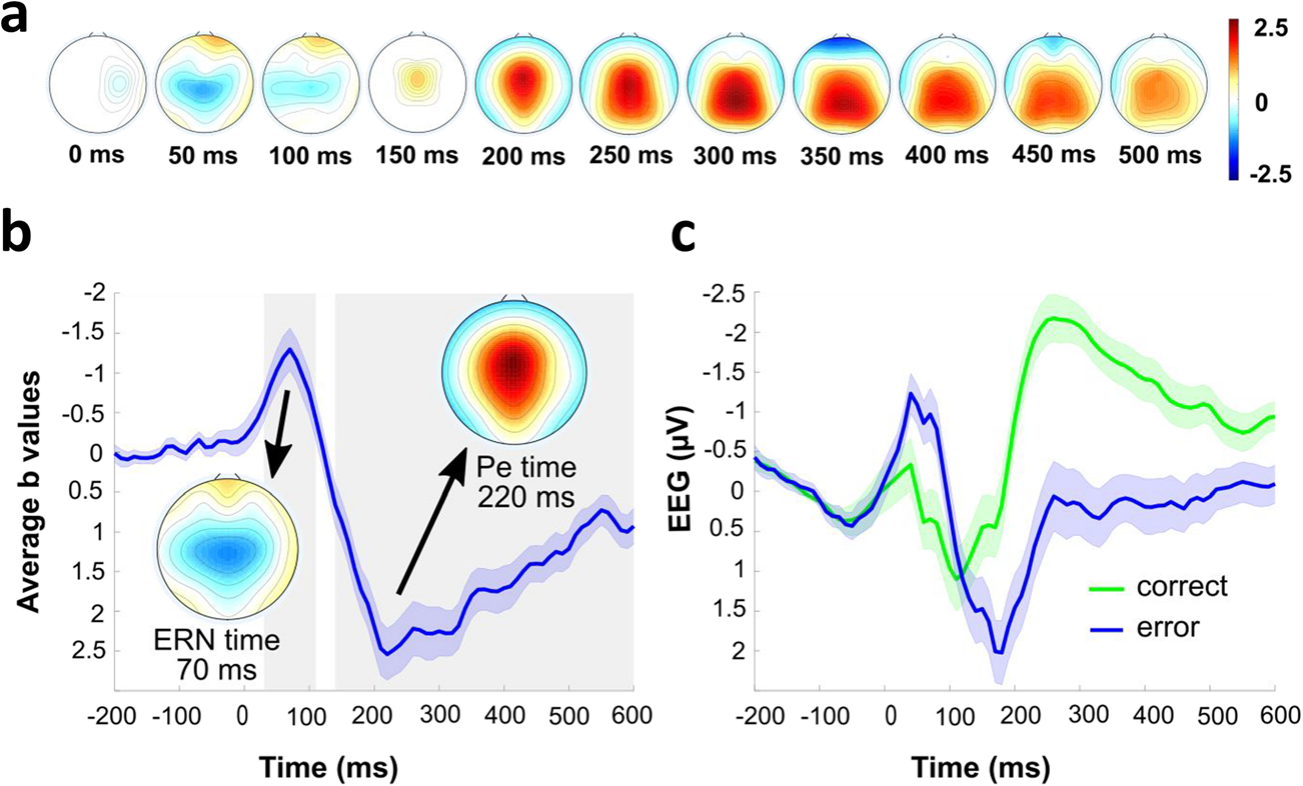
Error-related EEG activity. **A**) Regression weight topographies for the response-logged error regressor. Topographies show beta coefficients thresholded at critical *p*-value from FDR correction. **B**) Regression weights at electrode Cz. Gray shaded areas mark the significant time-points after FDR correction (Benjamini and Yekutieli, 2001). **C**) Regular ERPs at Cz, which do not account for error-unspecific task effects

First, we established whether general error processing is reflected in the EEG signal by submitting response-locked EEG epochs to multiple robust regression (Fischer et al., 2016; Fischer & Ullsperger, 2013). Scalp topographies of response-locked regression weights of the error regressor of EEG GLM 1 confirmed the typical ERN (Cz, peak at 70 ms, b = −1.30, 99% CI [−2.02, −0.60], *p*(corrected) = 9.87 x 10^-6^) and Pe succession (Cz, peak at 220 ms, b = −2.54, 99% CI [1.66, 3.42], *p*(corrected) = 1.87 x 10^-10^) after objective errors with maxima found at electrode Cz (Figure 4).

#### Modulation of error-related EEG activity by error awareness and confidence in subjective accuracy judgment

**Fig. 5:**
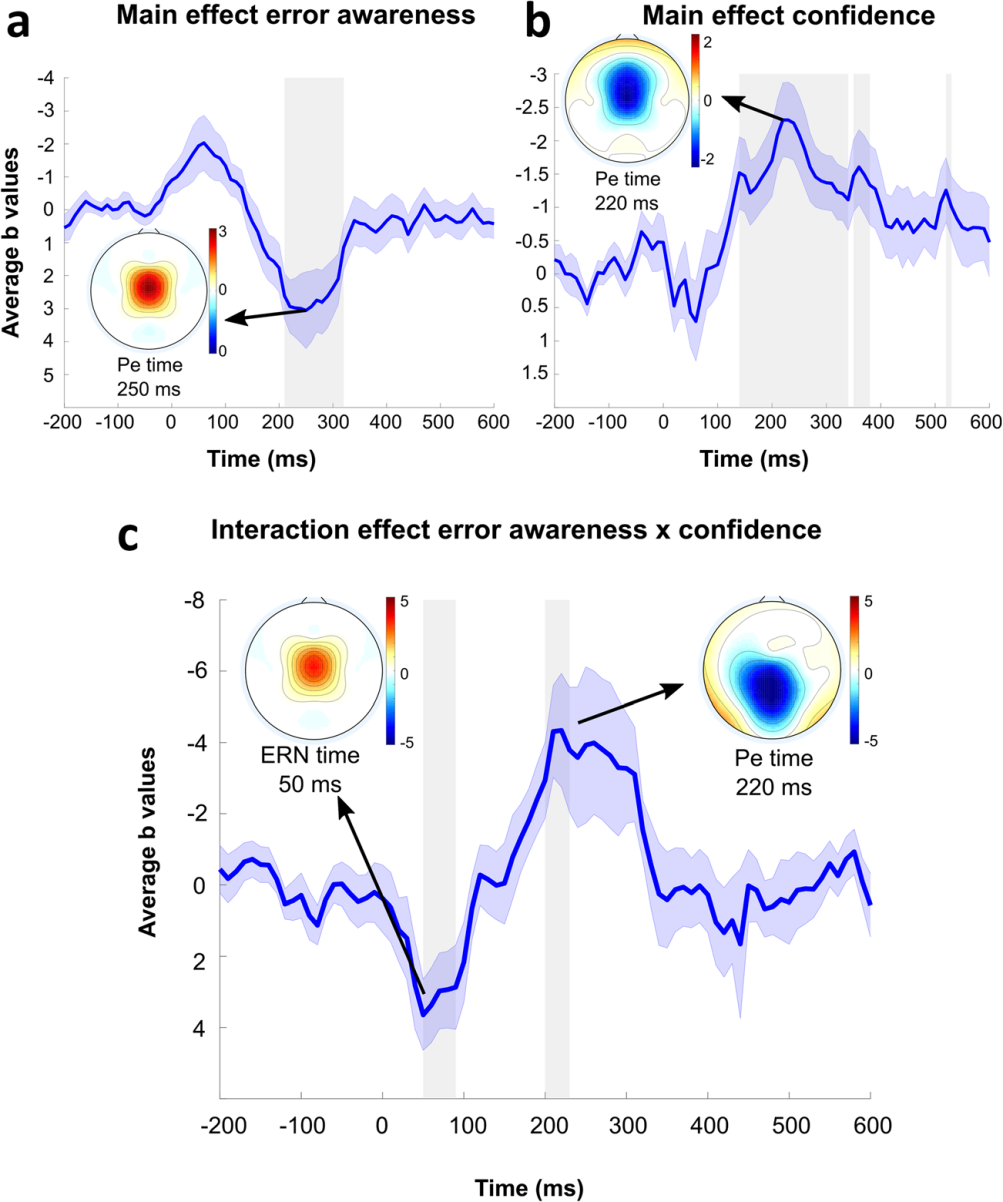
Modulation of error related EEG activity by error awareness and confidence in accuracy judgment*.* Regression weight time course and topographies for error awareness (**A**), confidence (**B**), and error awareness x confidence interaction (**C**). Topographies thresholded at 0.05/3 (alpha/number of regressors). Gray shades highlight the significant time points after correction for multiple comparisons

Second, we investigated if the ERN and Pe succession was modulated by error awareness and participants' confidence in their subjective accuracy evaluations (i.e., latency of the subjective error judgment). Regression analyses on response-locked error trials revealed that aware errors were associated with more negative EEG activity in the time range of the ERN, however this effect did not pass the correction for multiple comparisons (Cz, peak at 60 ms, b = −2.03, 99% CI [−4.29, 0.23], *p*(corrected) = 0.057). In addition, the error awareness regressor indicated that aware errors were associated with more positive activity in the time range of the Pe (Cz, peak at 250 ms, b = 3.05, 99% CI [−0.09, 6.19], *p*(corrected) = 0.006; Figure 5A). The confidence regressor revealed a negative covariance with the EEG signal in the time window of the Pe (Cz, peak at 220 ms, b = −2.31, 99% CI [−3.80, −0.82], *p*(corrected) < 0.001; Figure 5B). The effect represented smaller Pe amplitudes in trials in which participants took longer to make their accuracy judgment. Importantly, the error awareness effect depended on the factor confidence (Figure 5C). In the time window of the ERN, the error awareness x confidence interaction regressors covaried positively with the neural activity (Cz, peak at 50 ms, b = 3.37, 99% CI [0.53, 6.22], *p*(corrected) < .001). In addition, there was a negative covariation between the awareness x confidence interaction and the EEG signal in the time range of the Pe with a slightly earlier peak at Cz (peak at 220 ms, b = -4.34, 99% CI [-8.65, -0.04], *p*(corrected) = 0.005). These results indicate, that on error trials that were consciously perceived and where the accuracy judgment was made quickly, both the ERN and Pe were enhanced. A possible confound of these effects is that the trial type may influence ERN and Pe variations across aware and unaware errors. We ensured that all the effects from EEG GLM 2 could not be explained by this confound via the inclusion of the regressor Trial Type into the regression model (see supplementary Figure 4). Moreover, the time to prepare for the stimulus (i.e., the jitter) also may have influenced the crucial regressors in EEG GLM 2. When including the jitter regressor in the model, we did not see any systematic temporal or special influence of the jitter on the ERN or Pe amplitudes (see supplementary Figure 6). Interestingly, controlling for the jitter appears to have pushed the ERN effect within the error awareness regressor over the multiple comparison significance threshold (Cz, peak at 60 ms, b = −2.25, 99% CI −3.95, −0.53], *p*(corrected) = 0.036).

#### Error awareness prediction based on EEG signal

**Fig. 6:**
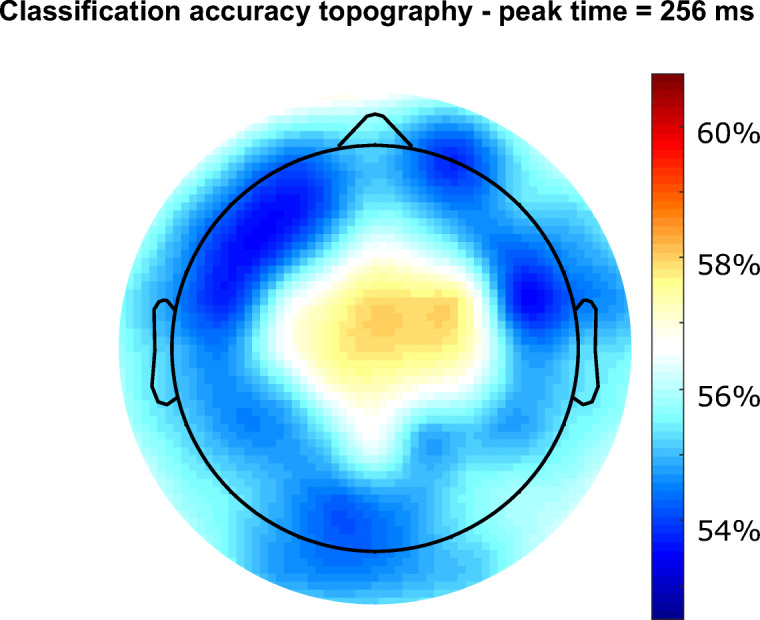
Error awareness prediction based on EEG signal

Next, we investigated the relationship between single-trial EEG activity and the subsequent accuracy judgment. For this, we used multivariate pattern analyses on single trial neural activity of the whole scalp to train a support vector machine to predict if a participant consciously perceived an error on a given trial. We found that the neural responses to errors were sufficient to predict whether or not a participant consciously perceived an error with an accuracy of 63.83% (peak at 258.42 ms, chance = 50%, permutation test *p* = 2x10^-5^). A searchlight analysis of the scalp distribution of this information was in accordance with the Pe topography (Figure 6).

#### Coupling between error-related EEG activity and post-error adjustments

**Fig. 7:**
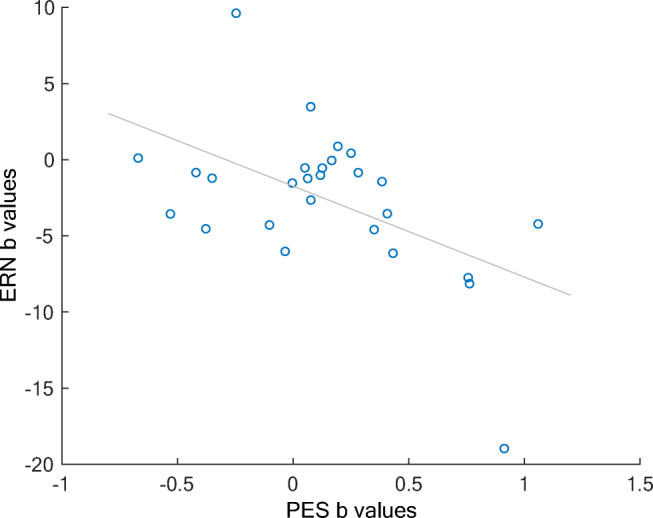
Correlation between individual post-error slowing and ERN regression weights

We first sought to investigate a possible relationship between single-trial neural activity and subsequent behavioural post-error adjustments seen in the behavioral analyses. To this end, we again regressed error-related EEG activity on a given trial onto reaction times of the following trial, while controlling for possible confounds (RT of the accuracy judgment, the consecutive trial’s type, congruence, and jitter). The results of this analysis revealed no significant covariance between the EEG signal and consecutive RT for either aware or unaware errors (all corrected *p* > 0.05). Next, we looked at a possible relationship between post-error slowing and the neural correlates of error awareness on a group level. Therefore, we correlated the regression weights of the ERN and Pe peak of the error awareness regressor for each participant with the respective regression weights of post-error slowing (PES) factor after aware errors. There was a significant negative correlation between the regression weights of the ERN and the PES factor (*r* = −0.39, *p* = 0.045; Figure 7), indicating a stronger coupling between the ERN and PES in individuals with higher ERNs after aware errors. There was no such relationship between PES and the regression weights of the Pe peak (*r* = 0.001, *p* = 0.97).

#### Differences in intertrial phase clustering between aware and unaware errors

**Fig. 8:**
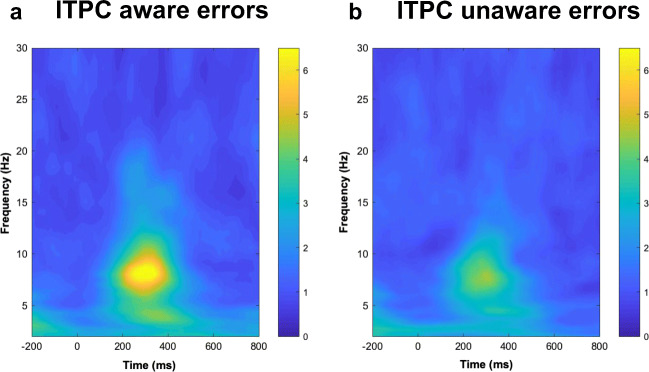
Intertrial Phase Clustering (ITPC) from electrode Cz over time-frequency space for aware (**A**) and unaware (**B**) errors. Higher values indicate higher phase clustering

To investigate whether latency variability could potentially contribute to the amplitude differences seen in the EEG analyses, we calculated the intertrial phase clustering (ITPC) as a measure of the consistency of time-frequency phase angles over trials (Cohen, 2014). The results of this analysis are depicted in Figure 8 and show that in the time window of the Pe, compared with unaware errors, aware errors induce stronger intertrial phase clustering in the theta to alpha frequency range. This indicates that in the time range of the Pe, this specific frequency-band activity is taking on a similar temporal configuration after the commission of aware errors. This seems to indicate that the larger Pe amplitude on aware errors may, in part, result from stronger time-locking of the underlying neuronal activity to the erroneous response. This argument is supported by a significant correlation between the regression weight time course of the error awareness regressor from EEG GLM 3 and the time course of the difference between ITPC for aware and unaware errors at 9 Hz^1^ (*r* = 0.34, *p* < 0.001).

## Discussion

In this study, we developed a number judgment error awareness task, which represents a new error awareness task based on complex rule representations similar to the error awareness task by Hester et al. (2005). With this task, we aimed at studying electrophysiological correlates of performance monitoring (ERN and Pe) and post-error behavioral adjustments (PES, PIA, and PERI) in relation to conscious error perception and participant’s confidence in the accuracy judgment. First, we showed that the task is well suited to study error awareness, because it yielded both a sufficient amount of aware and unaware errors. In contrast to a similar error awareness task developed by Hester et al. (2005), this new task has the advantage that it is not a Go/NoGo task, but individuals have to respond in every trial; therefore, comparing trials with and without a motor response can be avoided. In the following, we will first discuss the electrophysiological and behavioral results in the context of the current literature. We will then discuss the coupling between error-related EEG activity and post-error adjustments. Finally, we will conclude with a discussion on how error awareness relates to meta-control and outline avenues for future research on this topic.

### Neural traces of error awareness

Our electrophysiological results showed a modulation of both ERN and Pe by error awareness and confidence. The Pe was larger after aware errors as compared to unaware errors, which is in line with previous studies reporting the same effect (Dhar, Wiersema and Pourtois, 2011; Endrass et al., 2005; Murphy et al., 2012; Nieuwenhuis et al., 2001; O'Connell et al., 2007; Overbeek et al., 2005; Shalgi et al., 2009). For a long time, the dominant view has been that the ERN is not modulated by error awareness (Nieuwenhuis et al., 2001). However, in the present study, there was a tendency for a higher amplitude of the ERN for aware than for unaware errors. This error awareness influence on the ERN has been shown in other studies in an oculomotor task (Wessel et al., 2011), in a working memory task (Hewig, Coles, Trippe, Hecht and Miltner, 2011), and in paradigms that evoke unperceived errors by increasing the difficulty of detecting stimuli, either by employing degraded visibility of stimuli (Scheffers & Coles, 2000) or by metacontrast masking (Steinhauser & Yeung, 2010). Our results corroborate the findings by Shalgi and Deouell (2012), in that the ERN amplitude is also modulated by error awareness in complex task sets. Importantly, however, the effect of error awareness on both the ERN and Pe was dependent on participants' confidence in the accuracy judgment (i.e., the latency of the subjective accuracy judgment). Our data suggest, that on consciously perceived error trials where the accuracy judgment was made quickly, both the ERN and Pe were enhanced. These results are consistent with the findings of Boldt and Yeung (Boldt & Yeung, 2015), suggesting that the ERN and especially the Pe are correlates of error awareness and at the same time serve as a neural index associated with confidence in subjective accuracy judgments. Therefore, our results provide further evidence that error detection and decision confidence share neural markers. It should be noted that we only indirectly assessed confidence via RT of the accuracy judgment. Although RT has been suggested as a proxy for decision confidence (Kiani, Corthell and Shadlen, 2014), our results should be replicated in tasks that use more traditional confidence measurements, like wagering approaches (Persaud, McLeod and Cowey, 2007), or more fine graded decision confidence measurements similar to the Boldt and Yeung (2015) study, where participants expressed their decision confidence on a 6-point scale ranging from certainly wrong to certainly correct.

### Error awareness prediction based on EEG signal

The modulation of both the ERN and Pe amplitudes by error awareness and confidence, gives rise to the important question, which of these two neural error processing correlates serves as a more reliable precursor of error awareness. To this end, we used multivariate pattern analyses on single trial neural activity of the whole scalp to train a support vector machine to predict whether a participant consciously perceived an error on a given trial. A peak classification accuracy of ~63% in the time range of the Pe with a matching central topography suggests this component to be most predictive of error awareness. These results replicate previous work suggesting the Pe to be reliably predictive of error awareness (Boldt & Yeung, 2015; Steinhauser & Yeung, 2010).

Interestingly, an exploratory analysis of differences in intertrial phase clustering between aware and unaware errors suggest that in the time range of the Pe, neural activity in the theta to alpha band is taking on a more similar temporal configuration after the commission of aware errors. This seems to indicate that the larger Pe amplitude on aware errors may, in part, result from stronger time-locking of the underlying neuronal activity to the erroneous response. This increased consistency of time-frequency phase angles may contribute to the different neural correlates seen between aware and unaware errors.

### A global network may be responsible for conscious error detection

Taken together, our electrophysiological results are in line with Wessel (Wessel, 2012), who suggested that the ERN and possibly also the early Pe serve as feed-forward input signals into a more general system responsible for error awareness. Interestingly, recent research showed that the ERN is not always necessary for emergence of the Pe and error awareness (Charles, Van Opstal, Marti and Dehaene, 2013; Di Gregorio, Maier and Steinhauser, 2018; M. E. Maier, Di Gregorio, Muricchio and Di Pellegrino, 2015). This is seen for example in situations, where one detects an error without knowing the correct response (Charles et al., 2013; Di Gregorio et al., 2018), or in patients with compromised ERN (Maier et al., 2015). Thus, in line with Wessel (2012), the ERN may be one of many input signals to a global neural network to responsible for conscious error detection. The modulating effect of confidence in the accuracy judgment on the error awareness effect on both the ERN and Pe found in our study support the idea that these signals reflect a cumulative input of error evidence into this system (Murphy, Robertson, Harty and O'Connell, 2015).

### Behavioral traces of error awareness

On a behavioral level, the results in our number judgment error awareness task are typical for classical choice reaction time tasks and reflect effects of interference (incongruency, numerical distance to the reference number) and the interval between the last response and next on participants behavior. Moreover, trial type (standard vs. target) had an influence on accuracy and RT. Importantly, while controlling for these influencing variables, we investigated possible post-error adjustments in their relation to error awareness.

In the present study, post-error slowing (PES) appeared to be modulated by error awareness. While controlling for confounds and the interdependence of effects, we only found PES after aware errors. Previous studies investigating whether PES is modulated by error awareness showed mixed results: while some studies did not find such effects or even reported post-error speeding following aware errors (Hester et al., 2005; Hester et al., 2012; Logan and Crump, 2010; Orr & Hester, 2012), other studies reported greater PES following aware errors compared to unaware errors (Cohen et al., 2009; Endrass et al., 2007; Nieuwenhuis et al., 2001; Wessel et al., 2011). Our results suggest that conscious error perception is necessary to elicit PES. With respect to other post-error adjustments, we found no post-error reduction of interference (PERI) after both aware and unaware errors. Instead, our data suggests a general increase of the interference effect that is present only after aware errors. Given the small overall interference effect in our task, this finding is difficult to interpret. This effect might reflect a general slowing after aware errors, which is further enhanced by interference on the current trial. Moreover, while controlling for confounds on accuracy in the task, we did not find post-error increases of accuracy (PIA), neither after aware nor after unaware errors. This is in contrast to Klein et al. (2007), who reported PIA after aware but not after unaware errors. Notably, descriptively we do see increases in accuracy both after aware and unaware errors (~3-5%) but this effect might be better accounted for by the current trial type (i.e., most errors occurred on target trials, which are widely spaced in time during the experiment and most of the time were followed by standard trials). These results highlight the need to account for possible confounds in paradigms where post-error trials can require different cognitive processes as the trial before, where the error has been committed. Importantly, it should be noted, that we do not find differences in post error adaptations, when directly comparing behavior following aware and unaware errors (see additional GLM in Supplementary Figure S4). Hence, our conclusion about behavioral traces of error awareness should interpreted with caution and followed up in larger sample.

### Coupling between error-related EEG activity and post-error adjustments

Finally, we sought to investigate a possible relationship between single-trial neural activity on aware versus unaware errors trials and subsequent behavioral post-error adjustments, seen in the behavioral analyses. Here, we were not able to predict RT on the consecutive trial based on the error-related EEG activity in the current trial. However, on a group level, we found a stronger coupling between the ERN and post-error slowing (PES) in individuals with higher ERNs after aware errors that was not seen for the Pe. This across-participants correlation suggests that the better an individual’s performance monitoring system is in consciously detecting errors, the more likely they are to slow down after these aware errors. While the latter finding in general fits well with previous work showing that the ERN amplitude co-varied with PES (Di Gregorio, Steinhauser and Maier, 2016; Fischer et al., 2016; Gehring et al., 1993), the lack of a coupling between single-trial ERN and PES are at odds with studies showing this association on a single-trial level (Debener et al., 2005; Fu et al., 2019). This discrepancy might be explained by the prolonged response-stimulus interval caused by the secondary task (i.e., the accuracy judgment) in the current paradigm. Moreover, the fact that we used the same response modality for the error judgment also may have influenced post-error reaction times. Still, the global association between the larger ERN amplitudes and PES on aware error trials support the notion that behavioral adjustments are triggered by the ERN amplitudes and that the MFC consequently signals necessity for behavioral adjustments (Kerns et al., 2004; Ullsperger, Danielmeier and Jocham, 2014). Interestingly, our data indicate that error awareness may play a mediating role in this process. Critically, future studies should attempt to replicate our findings in a task with closer temporal proximity between responses. For example, the reliable link between error awareness, decision confidence, and the Pe in our study and previous work (Boldt & Yeung, 2015; Steinhauser & Yeung, 2010) suggest that this neural marker might be a promising index of error awareness and confidence, which could potentially replace disruptive secondary tasks, assessing error awareness in the task at hand. Another possibility would be to use a different response modality for indicating errors compared with the main task responses. One could for example consider using a verbal forced-choice error signaling procedure. Such an error signaling procedure would be less likely to influence manual post-error reaction times and measures of post-error behavioral adjustments.

### How does error awareness relate to meta-control?

Does our study provide insights into the putative role of conscious representations of performance errors and meta-control? In an ever-changing world, it is important to adjust control parameters, such as exploration versus exploitation, goal shielding and shifting, and short-term versus long-term goals to optimize goal-directed behavior (Goschke, 2013; Goschke & Bolte, 2017). When cognitive control is recruited by an adaptation signal from the performance monitoring system (Shenhav, Botvinick and Cohen, 2013; Ullsperger, Danielmeier and Jocham, 2014), two control modes can be distinguished, depending on whether it is “reactive” in nature, directed at resolving performance problems *ex post facto* in a transient manner, or “pro-active,” focused on preventing interference *ex ante facto* in a preparatory fashion (Braver et al., 2007; Ridderinkhof, 2002; Ridderinkhof et al., 2011). Post-error slowing, particularly in the absence of attentional focusing and performance improvement, has been suggested to represent reactive control (King et al., 2010; Notebaert et al., 2009; Ullsperger and King, 2010). As discussed earlier, our data support previous findings that post-error slowing is more likely and stronger after errors that reached conscious awareness than errors that remained unnoticed. This suggests that reactive control is implemented more strongly when the performance problem is consciously detected. It remains to be shown whether conscious error perception and reactive control are linked causally or whether both are induced in parallel and more strongly, when the objective evidence for errors (reflected, e.g., in the amplitude of the ERN) is stronger. Post-error reduction of interference and, more generally, post-error improvement in accuracy result from top-down control mechanisms (Botvinick, Cohen and Carter, 2004; Danielmeier et al., 2015; Danielmeier & Ullsperger, 2011) and are considered examples of proactive control (King et al., 2010). Unfortunately, similar to other tasks previously used to study error awareness, the number judgment error awareness task used here failed to reveal proactive post-error adjustments and, therefore, cannot address the role of conscious error perception for strategic implementation of proactive control. To further investigate proactive post-error adjustments and their modulation by conscious error perception, tasks with robust interference effects, that can be modulated after errors, are needed.

### Conclusions and avenues for future research

In conclusion, the results of this number judgment error awareness task corroborate previous findings that show a dependence of the size of both the ERN and Pe on error awareness and confidence in the accuracy rating. In line with previous work, our data suggest that the Pe is most predictive of error awareness, strengthening its role as a robust neural index of error awareness. Therefore, it could be used in the future as nondisruptive index of error awareness to avoid asking participants for an accuracy judgement after each trial. Additionally, we found partial support for a mediating role of error awareness on the coupling between the ERN and behavioral adjustments in the following trial, potentially highlighting error awareness as an important mechanism of meta-control processes. Furthermore, our data highlight the need to account for confounds and interdependence of effects in complex tasks, which can require different cognitive processes from trial to trial and that single-trial regression techniques on both behavioral and neural data (Fischer et al., 2016; Fischer & Ullsperger, 2013) are well suited for this demand. Finally, we hope that our suggestions for modifications and amendments of the current task will help to optimize future paradigms designed to investigate the link between error awareness and post-error adjustments.

## Electronic supplementary material

ESM 1 (PDF 1673 kb)

## Data Availability

The data and materials of this study can be downloaded on the Open Science Framework at https://osf.io/327pz/. All processing and analysis scripts are available from the authors upon reasonable request.

## Footnotes

1 9Hz was the frequency with the highest difference between aware an unaware errors.
