# Supplementary material for: Neural and behavioral traces of error awareness
Source: Cogn Affect Behav Neurosci. 2020 Oct 6;21(3):573–91. doi: 10.3758/s13415-020-00838-w (PMC8208913; doi:10.3758/s13415-020-00838-w)
Supplement: Supplementary file 1 — (PDF 1673 kb) [file 13415_2020_838_MOESM1_ESM.pdf]

Neural and behavioral traces of error awareness

Kirschner et al.

Supplementary Results

Relationship between the task response, accuracy judgement accuracy judgement RT

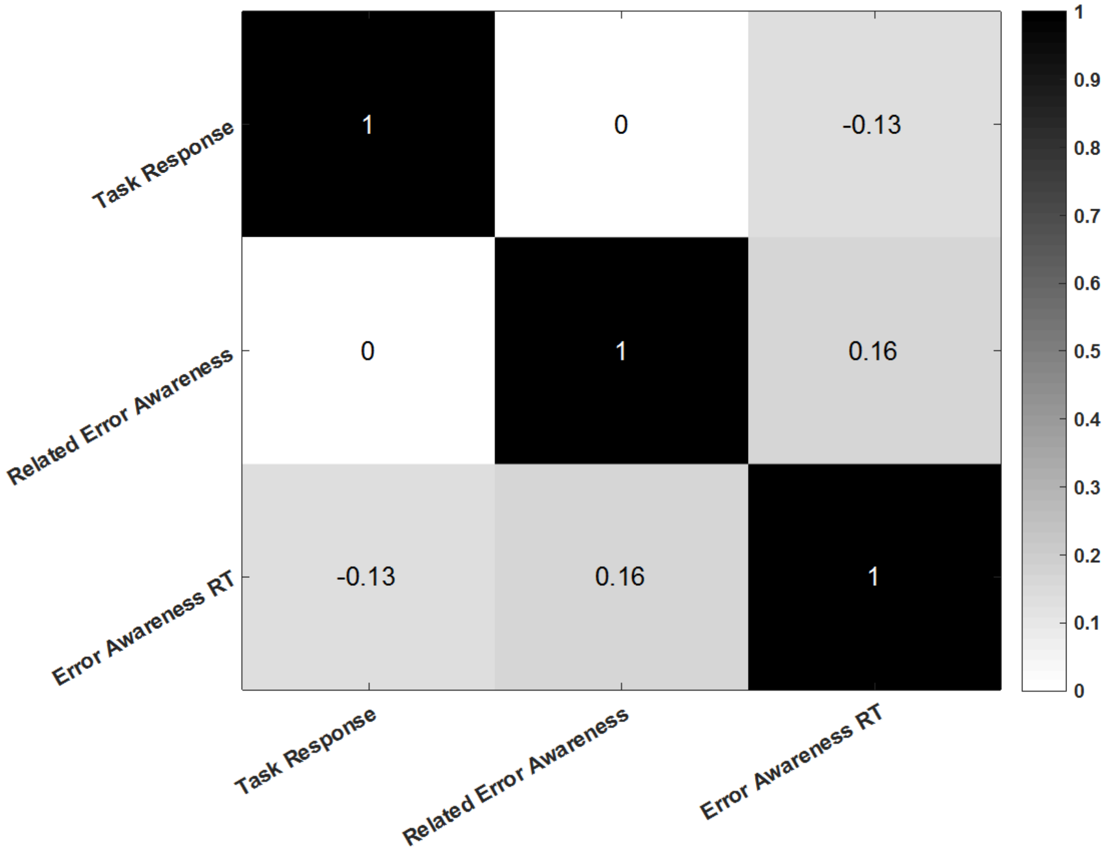

Supplementary Figure S1. Relationship between task response, accuracy judgment, and accuracy judgment RT. Correlation coefficients reflect the averaged within-subject correlation between the respective task parameters. Here, we do not see a systematic relationship between these task parameters. *Note*: the colorbar depicts absolute scores.

Detailed visualisation of the results of the RT and accuracy model

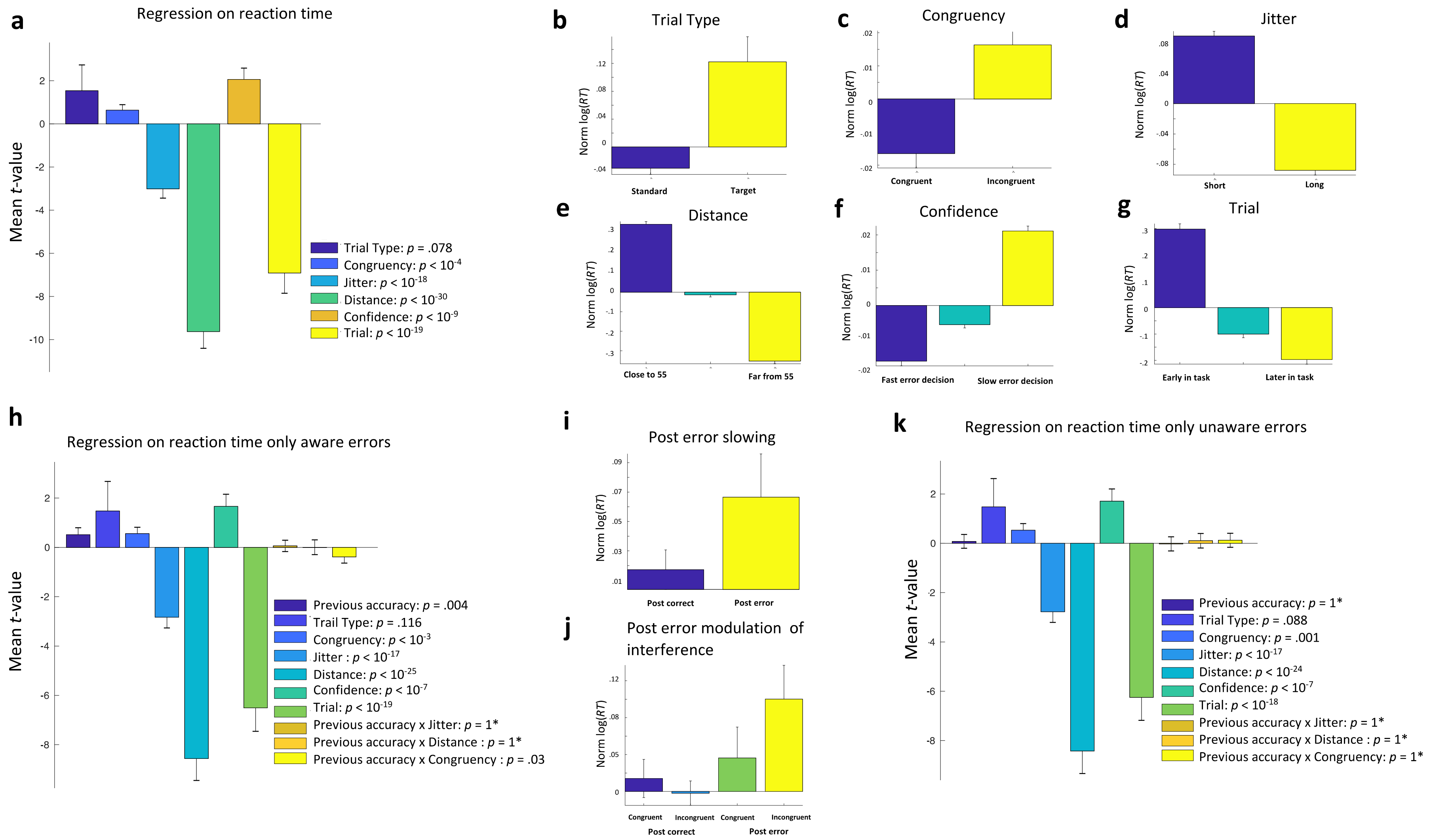

Supplementary Figure S2. Results of the RT regression model. **a** multiple single-trial regression on RT was used to evaluate general task behaviour. Results reflected an effect of trial type (standard vs. target; **b**), congruency (**c**), jitter (**d**), numerical distance to the reference number (**e**), confidence in the last response (**f**) and trial number (**g**) on RT in a given trial. Post-error adjustments in RT and its modulation by error awareness were analysed using the same multiple single-trial regression approach. Here the data was split into aware errors (**h**) and unaware errors (**k**). Results suggested PES was only observed after aware errors (**k**, **i**). Additionally, we found a modulation of the interference effect after aware errors (**j**). **a**, **h**, and **k** display averaged within subject *t*-values, *p* values are corrected for multiple comparison **(**0.05/number of regressors) and derived from *t*-test of individual against zero. * = after correction. Error bars represent ± 1 SD. Data of parametric regressors were split into three bins. *N* = 5 participants were excluded because no model fit was found.

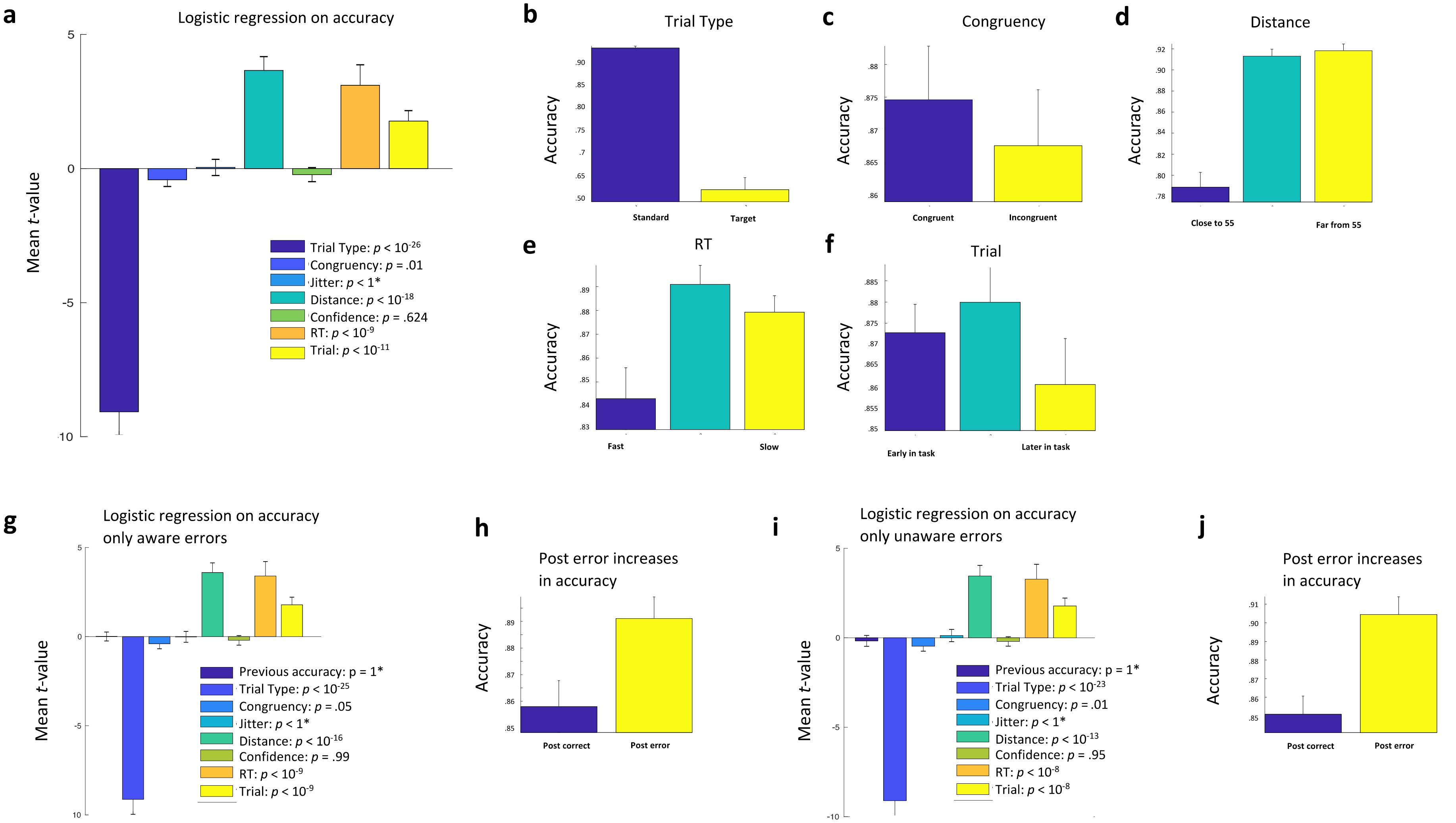
a
Logistic regression on accuracy
Mean t-value
Trial Type: p < 10-26
Congruency: p = .01
Jitter: p < 1*
Distance: p < 10-18
Confidence: p = .624
RT: p < 10-9
Trial: p < 10-11
b
Trial Type
Accuracy
Standard
Target
c
Congruency
Accuracy
Congruent
Incongruent
d
Distance
Accuracy
Close to 55
Far from 55
e
RT
Accuracy
Fast
Slow
f
Trial
Accuracy
Early in task
Later in task
g
Logistic regression on accuracy
only aware errors
Mean t-value
Previous accuracy: p = 1*
Trial Type: p < 10-25
Congruency: p = .05
Jitter: p < 1*
Distance: p < 10-16
Confidence: p = .99
RT: p < 10-9
Trial: p < 10-9
h
Post error increases
in accuracy
Accuracy
Post correct
Post error
i
Logistic regression on accuracy
only unaware errors
Mean t-value
Previous accuracy: p = 1*
Trial Type: p < 10-23
Congruency: p = .01
Jitter: p < 1*
Distance: p < 10-13
Confidence: p = .95
RT: p < 10-8
Trial: p < 10-8
j
Post error increases
in accuracy
Accuracy
Post correct
Post error

Supplementary Figure S3. Results of the logistic Accuracy regression model. **a**, **g** and **i** logistic regression on accuracy. Again, these models display averaged within-subject *t*-values, *p* values are corrected for multiple comparison (0.05/number of regressors) and derived from *t*-test against zero. In general, accuracy on a given trial was influenced by trial type (**b**), congruence (**c**), numerical distance from the reference number (**f**), RT (**e**), and trial number. Although, descriptively there was an increase in accuracy after both aware (**h**) and unaware errors (**j**), when controlling for other factors influencing accuracy on a given trial these effects did not reach significance (**g**, **i**). * = after correction. Error bars represent ± 1 SD. Data of parametric regressors were split into three bins. *N* = 6 participants were excluded because no model fit was found.

Modulation of error-related EEG activity by error awareness and confidence in subjective accuracy judgment

A possible confound of the effects reported from EEG Model 2 is that the trial type may influence ERN and Pe variations across aware and unaware errors. We ensured that the effects from EEG GLM 2 could not be explained by this confound via the inclusion of the regressor Trial Type into the regression model (EEG GLM 3). As can be seen in supplementary figure S4, all effects remained significant.

$$\begin{aligned} \mathrm{EEG} = b_0 &+ Error\ Awareness \times b_1 + Confidence \times b_2 \\ &+ Error\ Awareness\ Confidence \times b_3 + Distance \times b_4 + log(RT) \times b_5 \\ &+ Congruency \times b_6 + Trial\ Type \times b_7 + Trial\ Nr \times b_8 + e \end{aligned}$$

EEG GLM 3

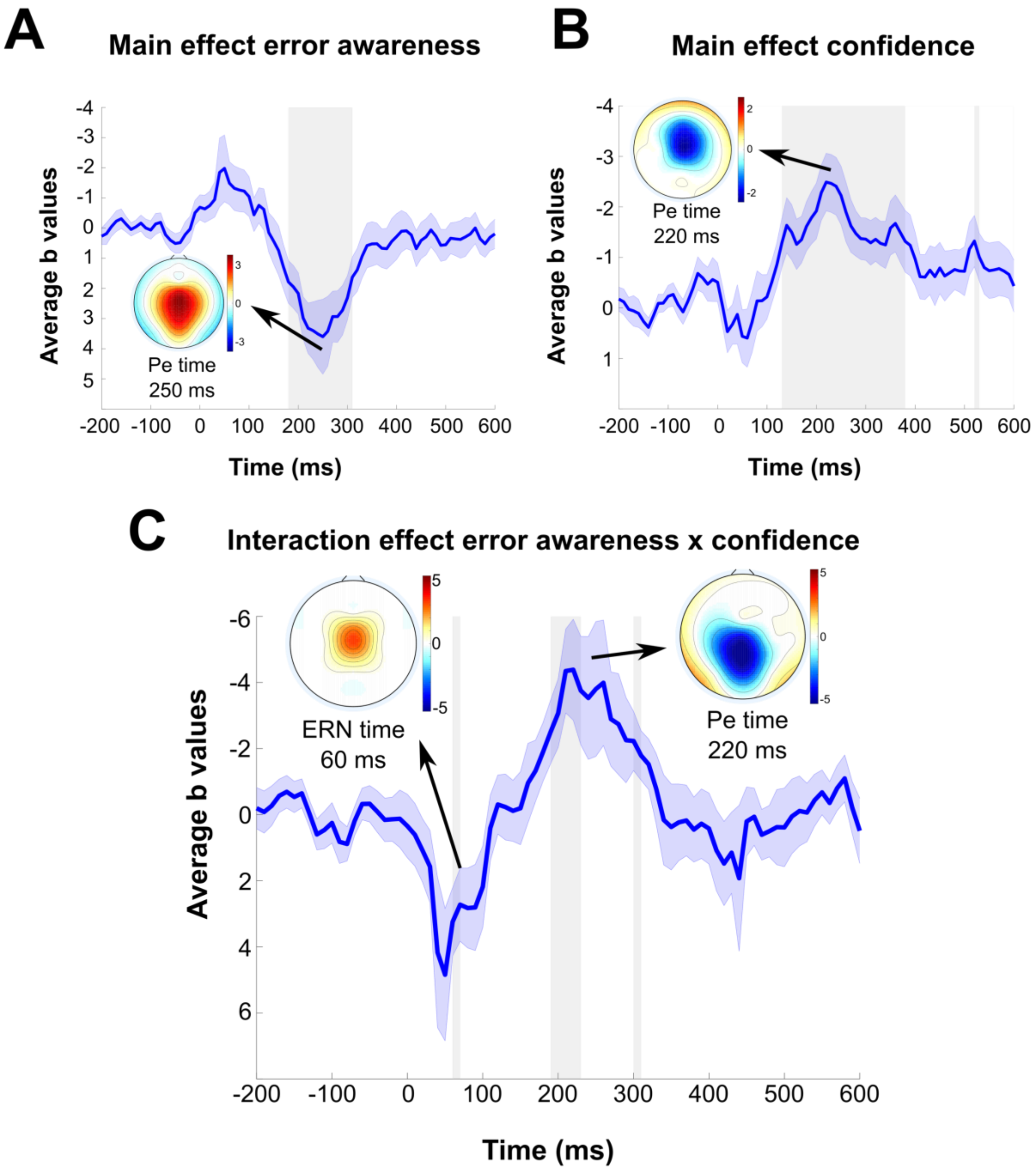

Supplementary Figure S4. Regression weight time course and topographies for EEG GLM 3. Topographies thresholded at 0.05/3 (alpha/number of regressors). Gray shades highlight the significant time points after correction.

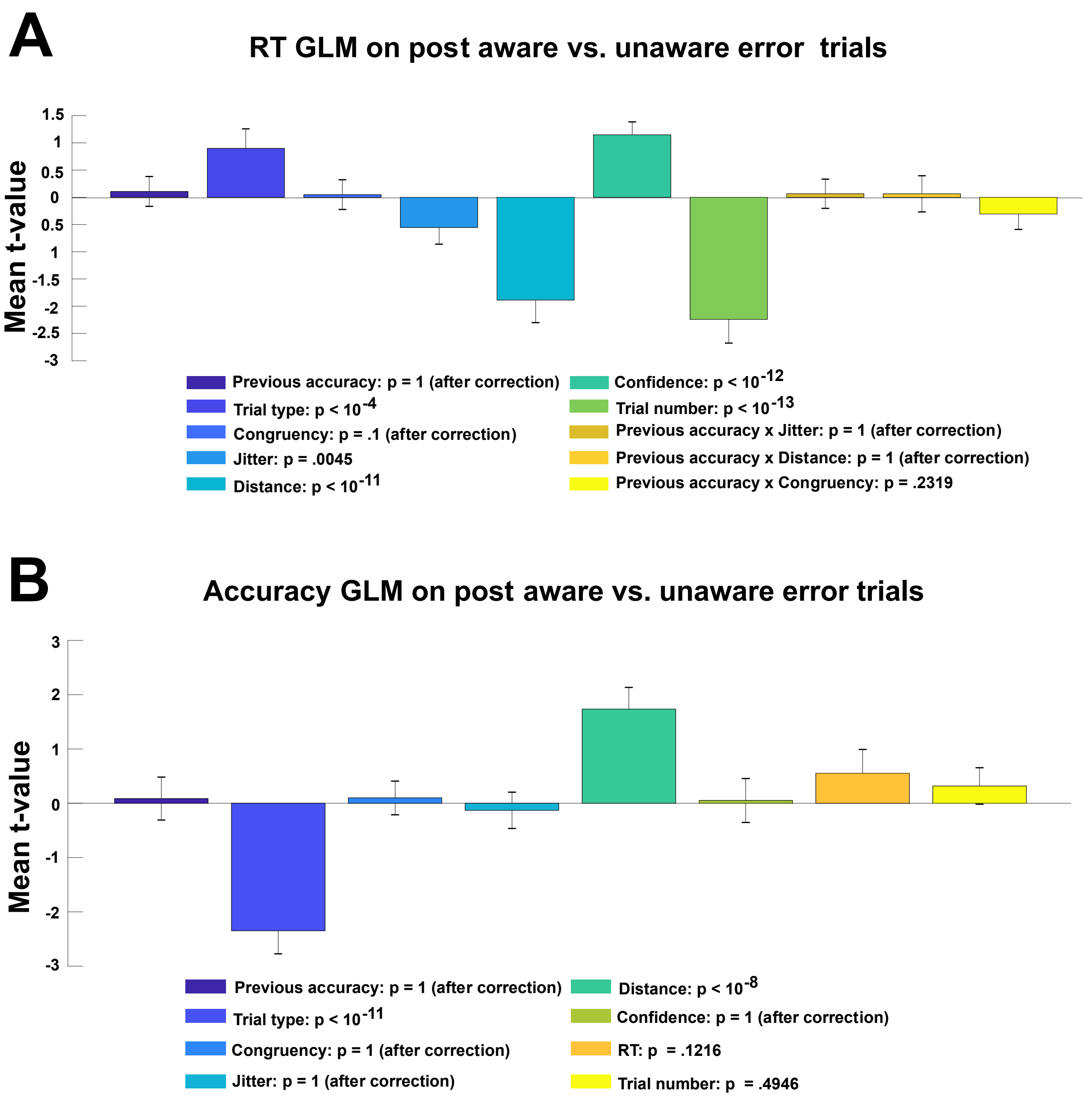

Supplementary Figure S5. Results of the RT and accuracy regression models directly comparing behavior following aware and unaware errors (excluding trials following correct trials). **A)** RT GLM on current correct and error trials post aware vs. unaware errors. **B)** Accuracy GLM on current correct and error trials post aware vs. unaware errors. Displayed are averaged within subject *t*-values, *p* values are corrected for multiple comparison (0.05/number of regressors) and derived from *t*-tests of individual t-values against zero. Results indicated that post error adaptations do not differ, when directly comparing behavior following aware and unaware errors.

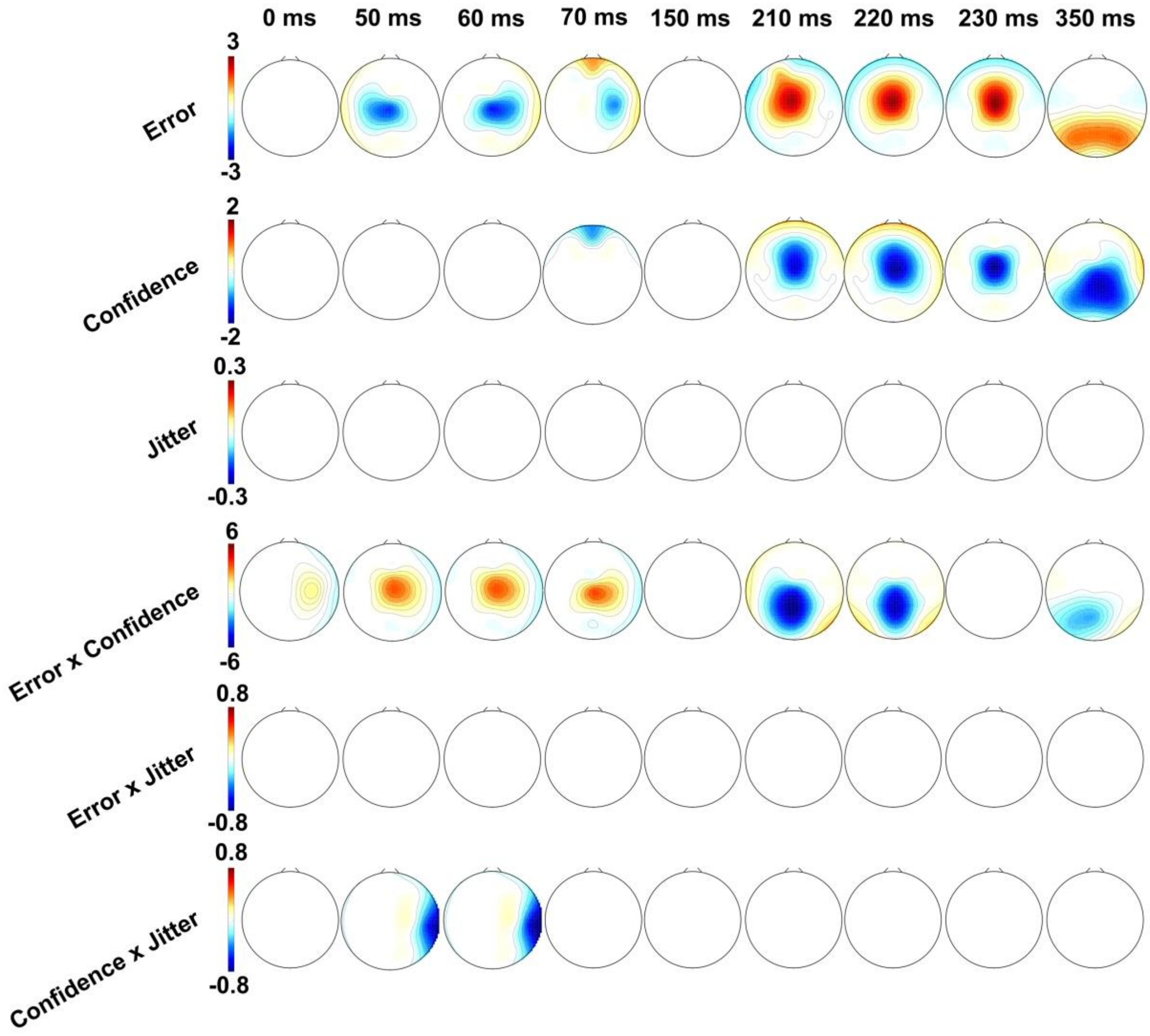

Supplementary Figure S6. Regression weight topographies of EEG GLM 2 including the additional regressor jitter. Topographies show beta coefficients thresholded at 0.05/3 (alpha/number of regressors in interest). Results indicate no systematic temporal or special influence of the jitter on the crucial regressors in our model.
